# NEU1-Mediated Extracellular Vesicle Glycosylation in Alzheimer’s Disease: Mechanistic Insights into Intercellular Communication and Therapeutic Targeting

**DOI:** 10.3390/ph18060921

**Published:** 2025-06-19

**Authors:** Mohd Adnan, Arif Jamal Siddiqui, Fevzi Bardakci, Malvi Surti, Riadh Badraoui, Mitesh Patel

**Affiliations:** 1 Department of Biology, College of Science, University of Ha’il, Ha’il P.O. Box 2440, Saudi Arabia; mo.adnan@uoh.edu.sa (M.A.);; 2 King Salman Center for Disability Research, Riyadh 11614, Saudi Arabia; 3 Research and Development Cell (RDC), Parul University, Waghodia, Vadodara 391760, Gujarat, India; 4 Department of Biotechnology, Parul Institute of Applied Sciences, Parul University, Waghodia, Vadodara 391760, Gujarat, India

**Keywords:** Neuraminidase 1 (NEU1), extracellular vesicles (EVs), glycosylation, Alzheimer’s disease, intercellular communication, glycomedicine

## Abstract

Alzheimer’s disease (AD), a progressive neurodegenerative disorder, is marked by the pathological accumulation of amyloid-β plaques and tau neurofibrillary tangles, both of which disrupt neuronal communication and function. Emerging evidence highlights the role of extracellular vesicles (EVs) as key mediators of intercellular communication, particularly in the propagation of pathological proteins in AD. Among the regulatory factors influencing EV composition and function, neuraminidase 1 (NEU1), a lysosomal sialidase responsible for desialylating glycoproteins has gained attention for its involvement in EV glycosylation. This review explores the role of NEU1 in modulating EV glycosylation, with particular emphasis on its influence on immune modulation and intracellular trafficking pathways and the subsequent impact on intercellular signaling and neurodegenerative progression. Altered NEU1 activity has been associated with abnormal glycan profiles on EVs, which may facilitate the enhanced spread of amyloid-β and tau proteins across neural networks. By regulating glycosylation, NEU1 influences EV stability, targeting and uptake by recipient cells, primarily through the desialylation of surface glycoproteins and glycolipids, which alters the EV charge, recognition and receptor-mediated interactions. Targeting NEU1 offers a promising therapeutic avenue to restore EV homeostasis and reduces pathological protein dissemination. However, challenges persist in developing selective NEU1 inhibitors and effective delivery methods to the brain. Furthermore, altered EV glycosylation patterns may serve as potential biomarkers for early AD diagnosis and monitoring. Overall, this review highlights the importance of NEU1 in AD pathogenesis and advocates for deeper investigation into its regulatory functions, with the aim of advancing therapeutic strategies and biomarker development for AD and related neurological disabilities.

## 1. Introduction

Alzheimer’s disease (AD) is a complex neurodegenerative disorder characterized by progressive cognitive decline and memory impairment, ultimately leading to severe dementia. AD is a significant global health issue, currently affecting over 55 million individuals worldwide, with this number expected to rise significantly due to increasing life expectancy and an aging population. It is the most common form of dementia among the elderly and projections estimate that the global burden may triple to approximately 139 million cases by 2050 [1]. AD presents not only a medical concern, but also a profound socio-economic challenge with global dementia-related costs exceeding $1.3 trillion annually, alongside an immense emotional toll on families and caregivers [2]. The progressive cognitive decline associated with AD often leads to a loss of independence, resulting in increased caregiver dependency and stress. This burden has far-reaching implications on healthcare systems, family structures and economic stability, highlighting the urgent need for comprehensive dementia care strategies and treatments [3,4,5,6].

The pathology of AD is marked by several key features, including the accumulation of amyloid-beta (Aβ) plaques, neurofibrillary tangles composed of hyperphosphorylated tau protein, neuroinflammation and synaptic dysfunction. These pathological hallmarks collectively contribute to neuronal loss and brain atrophy, which are central to the clinical manifestations of the disease [7,8,9]. The formation of Aβ plaques results from the cleavage of amyloid precursor protein (APP) by β-secretase and γ-secretase, leading to the aggregation of Aβ peptides into insoluble fibrils that deposit extracellularly in the brain [10,11]. These plaques are associated with neuroinflammatory responses, where activated microglia and astrocytes release pro-inflammatory cytokines and reactive oxygen species, further intensifying neuronal damage [8,12]. Additionally, neurofibrillary tangles, which consist of aggregated tau protein, are formed intracellularly and are believed to disrupt neuronal function, contributing to cell death [7,13]. The interplay between these pathological features highlights the multifaceted nature of AD and emphasizes the urgent need for novel therapeutic strategies targeting these mechanisms.

Current therapeutic approaches have largely focused on symptomatic relief rather than addressing the underlying pathology of AD. As such, there is an urgent need to develop innovative treatments that target the core pathological processes of the disease. Research is increasingly directed towards understanding the molecular mechanisms underlying Aβ production and tau phosphorylation, as well as the role of neuroinflammation in AD progression [9,14]. For instance, targeting the enzymes involved in Aβ production, such as β-secretase and γ-secretase, presents a promising avenue for therapeutic intervention [11]. Furthermore, exploring potential anti-inflammatory strategies to modulate microglial activation and reduce neuroinflammation could provide additional therapeutic benefits [8,15].

### 1.1. Extracellular Vesicles (EVs)

Extracellular vesicles (EVs) are a heterogeneous group of lipid-bound membrane structures released by cells into the extracellular space, playing crucial roles in intercellular communication. They are broadly classified into three subtypes, exosomes, microvesicles and apoptotic bodies, based on their mode of biogenesis pathways and physical characteristics. Exosomes, typically ranging from 30 to 150 nm in diameter, originate from the endosomal pathway and are released when multivesicular bodies (MVBs) fuse with the plasma membrane [16,17]. Microvesicles are larger (50–1000 nm) and formed by direct outward budding from the plasma membrane [18]. Apoptotic bodies, which can reach up to 5000 nm in size, are released during programmed cell death [18]. Despite their differences in origin, all EVs share the capacity to transport biologically active cargo between cells.

The molecular cargo of EVs includes proteins, lipids and nucleic acids, including messenger RNA (mRNA) and microRNA (miRNA), which reflect the physiological state of their parent cells [19,20]. Upon uptake by recipient cells, this cargo can modulate immune responses, cellular differentiation and tissue repair [21,22]. For example, EV-mediated transfer of signaling molecules facilitates dynamic intercellular communication, affecting processes like inflammation and tumor progression [23,24].

**Figure 1:**
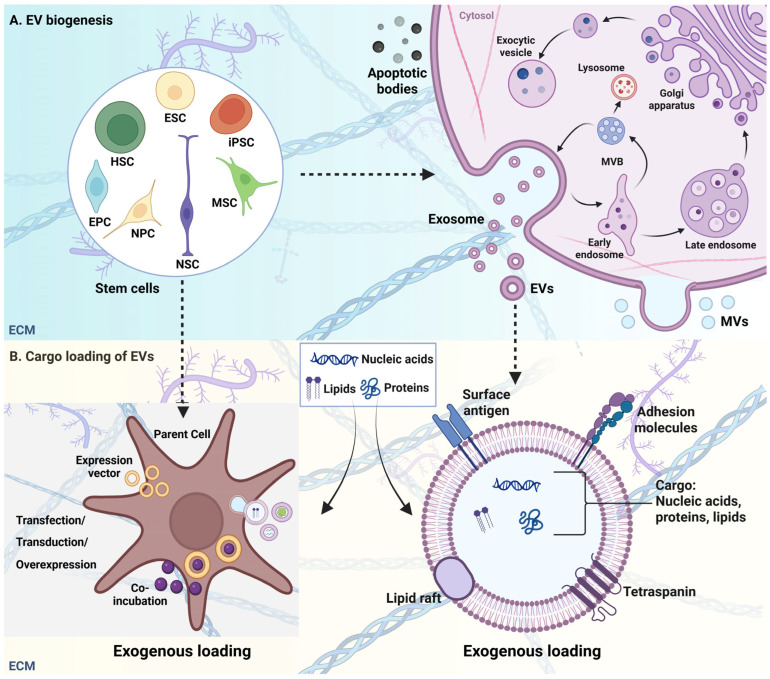
(**A**) EV biogenesis and origin from stem cell populations. This panel illustrates extracellular vesicle (EV) formation from various stem cell sources, including endothelial progenitor cells (EPC), hematopoietic stem cells (HSC), mesenchymal stem cells (MSC), neural stem cells (NSC), induced pluripotent stem cells (iPSC), embryonic stem cells (ESC) and neuronal progenitor cells (NPC). EVs are generated via two primary pathways: exosome formation, which begins with the inward budding of endosomes forming multivesicular bodies (MVBs) that fuse with the plasma membrane to release exosomes; and microvesicle (MV) formation, which involves direct outward budding of the plasma membrane. Additionally, apoptotic bodies arise during programmed cell death. (**B**) Strategies for bioactive cargo-loading into EVs. Endogenous loading involves genetic modifications of parental cells, including transfection, transduction or overexpression, resulting in the incorporation of therapeutic biomolecules (such as nucleic acids, lipids and proteins) during EV formation. Exogenous loading involves the direct incorporation of therapeutic molecules into isolated EVs via physical or chemical methods, such as co-incubation, electroporation or other mechanical approaches. The loaded EVs carry various bioactive cargos, including nucleic acids, lipids and proteins, which contribute to their therapeutic potential.

In the central nervous system (CNS), EVs are key mediators of communication between neurons and glial cells, contributing to synaptic plasticity, neuroprotection and the clearance of toxic proteins [25] (Figure 1). Notably, different EV subtypes may have distinct roles in neurodegeneration, as exosomes have been implicated in the intercellular spread of amyloid-beta (Aβ) and hyperphosphorylated tau. Microvesicles may regulate neuroinflammation through cytokine and lipid signaling, and apoptotic bodies contribute to the clearance of cellular debris but can also carry pro-inflammatory signals if not efficiently removed. These roles highlight the importance of EV heterogeneity in the progression and regulation of neurodegenerative diseases such as AD.

The importance of EVs in the CNS is highlighted by their potential roles in neurodegenerative diseases, such as AD, where they may participate in the spread of pathological proteins like amyloid-beta and tau [26]. Moreover, EVs can serve as biomarkers for various neurological conditions, providing insights into disease mechanisms and progression [19,20]. Their ability to cross the blood–brain barrier further enhances their therapeutic potential, making them promising candidates for drug delivery systems in CNS disorders [19].

### 1.2. Glycosylation and Its Importance

Glycosylation is a critical post-translational modification involving the enzymatic attachment of carbohydrate moieties to proteins, significantly influencing their structure, stability and function. There are two primary types of glycosylation, N-linked and O-linked. N-linked glycosylation occurs when glycans are attached to the nitrogen atom of asparagine residues within the consensus sequence Asn-X-Ser/Thr, where X can be any amino acid except proline [27,28]. This process typically begins in the endoplasmic reticulum (ER) and continues in the Golgi apparatus, where the glycan chains undergo extensive modification, including trimming and the addition of various sugar residues [29]. O-linked glycosylation, on the other hand, involves the attachment of glycans to the hydroxyl oxygen of serine or threonine residues, and it generally occurs in the Golgi apparatus [30]. Both types of glycosylation contribute to the vast diversity of glycoproteins, which can exhibit considerable heterogeneity in their glycan structures, influencing their biological roles.

The diversity of glycosylation is crucial for numerous biological processes. Glycosylation affects protein folding, stability and trafficking, thereby playing a vital role in determining protein function [31]. For example, N-linked glycosylation is essential for the proper folding of many glycoproteins, as it assists in the quality control mechanisms within the ER [28]. Additionally, glycosylation is pivotal in mediating cell–cell interactions and signaling pathways. Glycans on cell surfaces can serve as recognition sites for other proteins, facilitating communication between cells and influencing immune responses [32] (Table 1).

Moreover, glycosylation plays a significant role in the modulation of signaling pathways. Alterations in glycosylation patterns can affect the activity of signaling molecules, such as growth factors and receptors, thereby influencing cellular responses [33]. In cancer, for instance, changes in glycosylation can lead to enhanced tumor cell proliferation and metastasis, highlighting the importance of glycosylation in disease progression [34]. The interplay between glycosylation and signaling pathways, such as the Wnt/β–catenin pathway, illustrates how glycan modifications can have far-reaching effects on cellular behavior and fate [35]. Given the central role of glycosylation in modulating protein structure, signaling and immune recognition, alterations in glycosylation patterns have been increasingly linked to the pathogenesis of AD, particularly through their impact on amyloid precursor protein processing and tau protein stability.

**Table 1:** Glycosylation modifications in Alzheimer’s disease-associated proteins.

Gene	Glycosylation Type	Role in AD Pathology	Implication in EV Biology	References
TREM2	N-glycosylation	Altered N-glycosylation may influence ligand-binding affinity and contribute to AD pathogenesis.	Modulates microglial EV uptake and TREM2 shedding via EVs, influencing immune signaling	[36]
APOE	O-glycosylation	Changes in O-glycosylation may be associated with elevated Aβ_42_ levels and increased AD risk.	Affects APOE sorting into EVs and interaction with Aβ in EV-mediated transport	[37]
PSEN1	None	Does not undergo direct glycosylation but may regulate glycosylation of interacting proteins.	Impacts EV cargo composition indirectly via regulation of APP glycosylation	[38,39,40]
Nicastrin	N-glycosylation	Exists in both immature and mature glycosylated forms; the functional implications remain unclear.	Glycosylation state regulates its inclusion into EVs and ϒ-secretase activity in EVs	[41,42]
Tau	O-GlcNAcylation, N-glycosylation	O-GlcNAcylation is potentially neuroprotective but reduced in AD; N-glycosylation is observed specifically in AD conditions.	Affects tau stability and EV loading, potentially promoting trans-neuronal tau spread	[43,44,45,46,47]
BACE1	N-glycosylation	N-glycosylation, particularly with bisecting GlcNAc, enhances Aβ production and is upregulated in AD.	Modulates BACE1 secretion via EVs and contributes to extracellular Aβ generation	[48,49]
APP	N-glycosylation, O-glycosylation	Altered N-glycosylation impairs APP trafficking and processing; O-glycosylation may reduce Aβ secretion.	Regulates APP sorting into EVs, influencing Aβ production and propagation	[50,51,52]

(TREM2—Triggering receptor expressed on myeloid cells 2, APOE—Apolipoprotein E, PSEN1—Presenilin 1, APP—Amyloid precursor protein and BACE1—Beta-site APP cleaving enzyme 1).

### 1.3. Neuraminidase 1 (NEU1)

Neuraminidase 1 (NEU1) is a member of the neuraminidase family of enzymes, which are responsible for the hydrolysis of sialic acid residues from glycoproteins and glycolipids. NEU1 specifically catalyzes the removal of sialic acids from glycoproteins, playing a crucial role in various biological processes, including cell signaling, adhesion and immune responses [53,54]. The enzymatic activity of NEU1 is essential for maintaining the proper sialylation status of glycoproteins, which in turn affects their stability and function [54].

NEU1 is predominantly localized in lysosomes, where it participates in the degradation of sialylated substrates, but it can also be found on the cell surface [53]. This dual localization allows NEU1 to engage in both intracellular and extracellular functions. On the cell surface, NEU1 can trim sialic acid residues from glycoproteins, thereby modulating cell–cell interactions and influencing signaling pathways [53]. The enzymatic activity of NEU1 is particularly important in the context of immune responses, as the removal of sialic acids can expose underlying epitopes on glycoproteins, enhancing recognition by immune cells [53].

**Figure 2:**
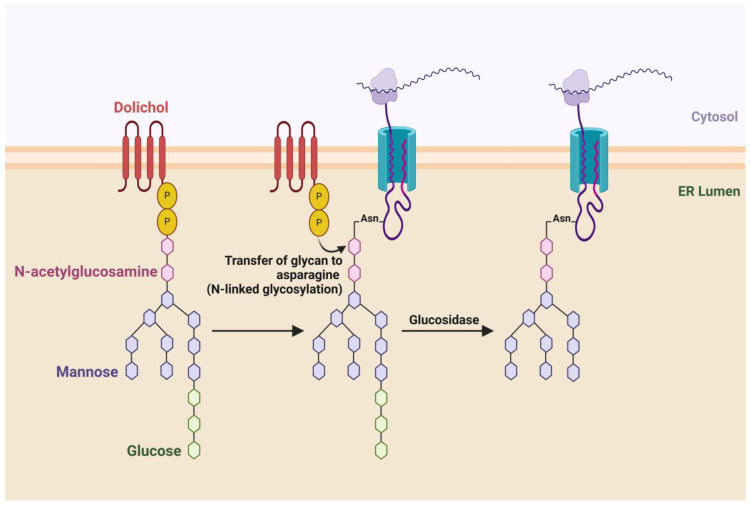
The process of N-linked glycosylation in the endoplasmic reticulum (ER). The glycan precursor, composed of N-acetylglucosamine, mannose and glucose, is assembled on a dolichol phosphate carrier embedded in the ER membrane. The oligosaccharyltransferase (OST) enzyme complex transfers the glycan moiety to a nascent polypeptide’s asparagine (Asn) residue, initiating N-linked glycosylation. Subsequently, glucosidases trim glucose residues from the glycan structure, facilitating proper protein folding and quality control within the ER lumen. This process is critical for protein maturation and trafficking.

The functions of NEU1 extend beyond simple desialylation. It has been implicated in various physiological processes, including the regulation of inflammation and the modulation of neuronal signaling [53] (Figure 2). For example, NEU1 has been shown to influence the sialylation of glycoproteins involved in neuronal communication, which is critical for maintaining synaptic function and plasticity [54]. Additionally, alterations in NEU1 activity have been associated with several pathological conditions, including neurodegenerative diseases and cancer, where dysregulation of sialylation can lead to altered cell behavior and disease progression [53,54].

Therefore, NEU1 is a vital enzyme that removes sialic acids from glycoproteins, with significant roles in lysosomal degradation and cell surface interactions. Its enzymatic activity is crucial for maintaining cellular homeostasis and regulating various biological processes, making it an important focus for understanding both normal physiology and disease mechanisms. The present review focuses on the specific role of NEU1 in modulating the glycosylation of extracellular vesicles within the context of AD. The major aim of the present review is to elucidate how NEU1 influences the glycosylation patterns of EVs and the subsequent implications for intercellular communication and the propagation of pathological processes associated with AD.

NEU1 is known for its enzymatic activity in removing sialic acids from glycoproteins and glycolipids, a process that significantly impacts the biological functions of these molecules [53,55]. In the context of AD, alterations in glycosylation, particularly the sialylation status of glycoproteins, can affect the release and composition of EVs, which are critical mediators of intercellular communication [56,57]. Thus, this review discusses how NEU1’s activity can lead to changes in the glycosylation profiles of EVs, potentially influencing their role in the spread of amyloid-beta (Aβ) and tau proteins, which are central to AD pathology [55]. Furthermore, information about the implications of NEU1-mediated glycosylation changes on the signaling pathways involved in neuroinflammation and neuronal communication is also presented. Given that EVs can carry misfolded proteins and other bioactive molecules, understanding the role of NEU1 in this context may provide insights into how AD propagates at the cellular level [56,58]. Furthermore, this review explores the potential of targeting NEU1 as a therapeutic strategy to modulate EV glycosylation and mitigate the progression of AD, thereby addressing the urgent need for novel interventions in this debilitating disease [59].

## 2. Extracellular Vesicles (EVs) in Alzheimer’s Disease Pathogenesis

Extracellular vesicles (EVs) play a significant role in the pathogenesis of AD by facilitating the spread of Aβ and tau pathology, contributing to neuroinflammation and inducing synaptic dysfunction. The ability to transport misfolded proteins and other bioactive molecules across cellular barriers underscores their importance in intercellular communication and disease progression. One of the primary ways by which EVs contribute to AD pathology is through the transference of Aβ and tau proteins. Studies have shown that EVs can carry aggregated forms of Aβ, which can induce neurotoxic effects in recipient neurons [60,61]. For example, Eitan et al. (2016) demonstrated that EV-associated Aβ mediates bioenergetic deficits and calcium handling impairments in neuronal models, suggesting a mechanism by which Aβ spreads its toxic effects [60]. Additionally, Polanco et al. (2016) reported that EVs isolated from the brains of tau transgenic mice can seed tau protein aggregation in a threshold-dependent manner, indicating that even small amounts of tau in EVs can significantly influence the propagation of tau pathology [62]. This prion-like behavior of Aβ and tau within EVs supports the hypothesis that AD pathology can spread through interconnected neuronal networks, exacerbating disease progression.

Moreover, EVs are implicated in neuroinflammation, a hallmark of AD. The release of inflammatory mediators from EVs can activate microglia and astrocytes, leading to a chronic inflammatory state that further contributes to neuronal damage [63,64]. For example, Crotti et al. (2019), found that microglial-derived EVs can promote tau spreading, highlighting the role of EVs in mediating inflammatory responses that facilitate the progression of tau pathology [65]. The interaction between EVs and the immune system is crucial, as it can modulate the inflammatory environment in the brain, potentially leading to increased neuronal vulnerability. In addition to their role in spreading pathological proteins and mediating inflammation, EVs also contribute to synaptic dysfunction in AD. Gabrielli et al. (2022) noted that EVs carrying Aβ can induce synaptic alterations, leading to impaired neurotransmission and synaptic loss [63]. The presence of soluble oligomeric forms of Aβ within EVs has been identified as a significant factor in synaptic toxicity, suggesting that EVs not only transport these proteins but also influence their pathological effects on synapses [63]. Furthermore, the interaction of EVs with synaptic components can disrupt normal signaling pathways, further exacerbating synaptic dysfunction and contributing to cognitive decline in AD patients.

Extracellular vesicles (EVs) play a pivotal role in the pathogenesis of AD by serving as carriers for various bioactive molecules, including Aβ oligomers, tau protein, inflammatory cytokines and other relevant components. The cargo of EVs is crucial for understanding their contributions to disease progression and intercellular communication in the context of AD (Table 2). Aβ oligomers are considered one of the most toxic species in AD and are frequently found within EVs. These oligomers can disrupt synaptic function and contribute to neurodegeneration [66,67]. Studies have demonstrated that EVs can transport Aβ oligomers from one cell to another, facilitating the spread of toxicity and contributing to the prion-like propagation of AD pathology [68]. The presence of Aβ in EVs enhances its bioavailability and influences the cellular environment, leading to synaptic dysfunction and neuronal death [69]. Tau protein, another critical player in AD pathology, is also found in EVs. The release of tau from neurons via EVs has been shown to promote tau aggregation and spread tau pathology to neighboring cells [70]. Research indicates that tau-containing EVs can seed the aggregation of tau in recipient neurons, thereby exacerbating neurodegeneration [62]. The interaction between tau and Aβ within EVs may further amplify the neurotoxic effects, creating a vicious cycle of pathology [67,70].

In addition to Aβ and tau, EVs are enriched with inflammatory cytokines that can modulate the neuroinflammatory response in AD. Microglia, the resident immune cells in the brain, release EVs containing pro-inflammatory cytokines, which can activate neighboring glial cells and perpetuate a chronic inflammatory state [63,64]. This neuroinflammation is a hallmark of AD and contributes to neuronal damage and cognitive decline [65]. The cytokine cargo of EVs can thus influence the overall inflammatory milieu in the brain, impacting disease progression. EVs also carry various other molecules, including lipids, nucleic acids (such as mRNA and miRNA) and proteins involved in cellular signaling and metabolism [69]. These components can modulate cellular responses and contribute to the dysregulation observed in AD. For example, changes in the lipid composition of EVs can affect their interaction with neuronal membranes, influencing the uptake of toxic proteins and altering synaptic function [75]. Furthermore, the presence of specific proteins in EVs may serve as biomarkers for disease progression, providing insights into the underlying mechanisms of AD [82].

## 3. EV Uptake and Targeting in the Brain

Extracellular vesicles (EVs) play a crucial role in intercellular communication within the brain, particularly in the context of AD. The uptake of EVs by various cell types, including neurons, microglia and astrocytes, is mediated by several mechanisms that contribute to disease propagation.

### 3.1. Mechanisms of EV Uptake

The uptake of EVs by recipient cells in the brain occurs through various endocytic pathways, including clathrin-mediated endocytosis, micropinocytosis and lipid raft-mediated uptake. Microglia and astrocytes have been shown to utilize macropinocytosis for the internalization of EVs, allowing them to engulf larger vesicular structures [83]. Neurons, while also capable of taking up EVs, may exhibit different uptake mechanisms influenced by the specific cargo and surface proteins present on the EVs [84]. Astrocytes have been reported to take up EVs through receptor-mediated endocytosis, where specific receptors on their surfaces recognize ligands on the EVs, facilitating their internalization [85]. Microglia, as proficient phagocytes, are particularly adept at engulfing EVs, which can lead to the activation of inflammatory pathways and modulation of their functional state [86]. The differential uptake mechanisms among these cell types highlight the specificity of EV interactions and their potential impact on cellular responses.

### 3.2. Contribution to Disease Propagation

The uptake of EVs by neurons, microglia and astrocytes contributes significantly to the propagation of AD pathology. For example, EVs containing Aβ oligomers can be internalized by neurons, leading to synaptic dysfunction and neurotoxicity [67]. The presence of Aβ in EVs enhances its bioavailability, allowing for the spread of toxic effects across neuronal networks [69]. Furthermore, tau protein released in EVs can seed aggregation in recipient neurons, intensifying tau pathology and contributing to neurodegeneration [70]. In microglia, the uptake of EVs can activate inflammatory responses, leading to the release of pro-inflammatory cytokines that further exacerbate neuroinflammation [87]. This inflammatory milieu can create a feedback loop, where activated microglia release additional EVs containing inflammatory mediators, prolonging the cycle of inflammation and neuronal damage [88]. The interaction between microglia and astrocytes is also critical; activated microglia can influence astrocytic function through EV-mediated signaling, leading to reactive astrocyte phenotypes that may either promote neuroprotection or contribute to neurotoxicity, depending on the context [89]. Astrocytes, through their uptake of EVs, can also modulate neuronal survival and function. For example, astrocyte-derived EVs containing neuroprotective factors can enhance neuronal resilience against oxidative stress [85]. However, in the context of AD, the balance may shift towards a more detrimental effect as reactive astrocytes release EVs that contain pro-inflammatory and neurotoxic factors [88].

## 4. Glycosylation of Extracellular Vesicles

A critical aspect of EV biology is their glycosylation, which involves the addition of carbohydrate moieties (glycans) to proteins and lipids on the EV surface. The types of glycans present on EVs significantly influence their properties, including stability, targeting and uptake by recipient cells. EVs are primarily decorated with two main types of glycans: N-linked and O-linked glycans. N-linked glycans are attached to the nitrogen atom of asparagine residues and are often complex structures that can include various modifications such as fucosylation and sialylation [90]. O-linked glycans, on the other hand, are attached to the hydroxyl group of serine or threonine residues and can also exhibit considerable diversity, including O-GalNAc and O-GlcNAc modifications [91]. The specific glycan profiles can vary depending on the cellular origin of the EVs and the physiological or pathological state of the producing cells.

The glycosylation of EVs has profound implications for their stability, targeting and uptake. Glycans can enhance the stability of EVs in circulation by providing a protective layer that shields the underlying proteins and lipids from degradation. For example, sialylated glycans can contribute to the longevity of EVs in the bloodstream, allowing for prolonged interaction with recipient cells [90,92]. The presence of specific glycans on EVs can dictate their targeting to particular cell types. For example, glycan-binding proteins (lectins) on recipient cells can recognize and bind to specific glycan structures on EVs, facilitating selective uptake [93] (Figure 2). This targeting is crucial in pathological contexts, such as cancer, where tumor-derived EVs may carry distinct glycan signatures that promote their interaction with tumor-associated cells [94].

The uptake of EVs by recipient cells is influenced by their glycan composition. Studies have shown that alterations in glycosylation can modulate the efficiency of EV uptake. For example, deglycosylated EVs exhibit enhanced cellular uptake, suggesting that glycosylation can prevent indiscriminate binding to proximate tissues [95]. Additionally, specific glycan structures can enhance the internalization of EVs through receptor-mediated endocytosis, impacting the biological responses of recipient cells [93]. In the context of AD, the glycosylation patterns of EVs can influence the propagation of pathological proteins such as Aβ and tau. For example, EVs enriched with specific sialylated or fucosylated glycans may facilitate the transfer of toxic oligomers between neurons, contributing to synaptic dysfunction and neurodegeneration [91]. Furthermore, changes in the glycosylation of EVs derived from glial cells can modulate inflammatory responses, thereby impacting the neuroinflammatory landscape associated with AD [90,92].

## 5. Techniques for Analyzing EV Glycosylation

**Figure 3:**
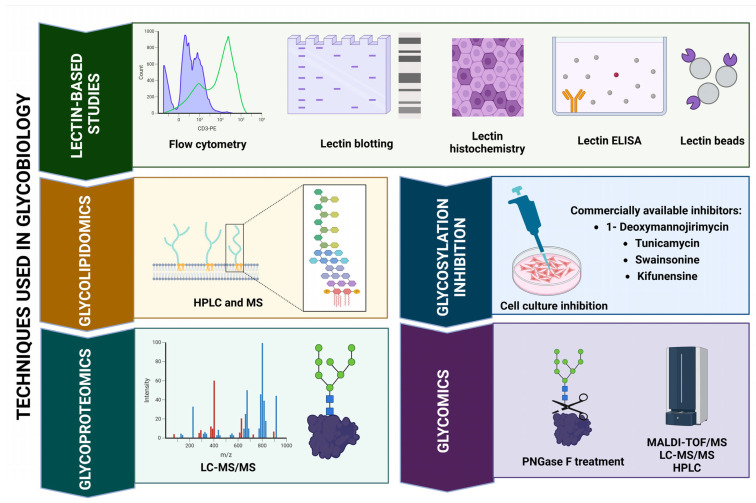
Illustration of major methodologies in glycobiology, including lectin-based studies for glycan detection, glycolipidomics (HPLC, MS) for glycosphingolipid analysis, glycoproteomics (LC-MS/MS) for glycoprotein characterization and glycomics (MALDI-TOF/MS, LC-MS/MS, HPLC) for glycan profiling. Glycosylation inhibitors, such as 1-deoxymannojirimycin, tunicamycin, swainsonine and kifunensine, are tools to study glycan biosynthesis and processing in cell-based systems.

Analyzing the glycosylation of EVs is crucial for understanding their biological functions and implications in various diseases, including AD. Several techniques have been developed to characterize the glycan profiles on EVs, each with its advantages and limitations (Figure 3).

### 5.1. Lectin Microarrays

Lectin microarrays are a powerful tool for profiling glycosylation patterns on EVs. This technique utilizes a variety of plant-derived lectins that specifically bind to different glycan structures, allowing for the simultaneous analysis of multiple glycan types [96,97]. The advantages of lectin microarrays include high-throughput capabilities, sensitivity and the ability to provide a comprehensive overview of glycan features without the need for prior glycan release from proteins. This method has been widely applied in cancer research to identify glycan changes associated with tumor progression and has potential applications in studying EVs derived from various cell types [96,97].

### 5.2. Mass Spectrometry (MS)

Mass spectrometry is another essential technique for analyzing EV glycosylation. It allows for the detailed characterization of glycan structures, including their composition, linkage types and branching patterns [96,97]. Techniques such as Matrix-Assisted Laser Desorption/Ionization Time-of-Flight (MALDI-TOF) and Electrospray Ionization (ESI) MS are commonly used to analyze glycoproteins and their glycan moieties [98]. Mass spectrometry can provide quantitative data on glycan abundance and is particularly useful for identifying specific glycan modifications that may influence EV function and interactions with recipient cells.

### 5.3. Enzymatic Digestion

Enzymatic digestion is often employed in conjunction with mass spectrometry to analyze the glycosylation status of EVs. For example, treatment with peptide N-glycosidase F (PNGase F) can remove N-linked glycans from glycoproteins, allowing for the analysis of the core protein structure and the identification of glycosylation sites [32]. This approach helps elucidate the functional significance of specific glycosylation patterns in EV biology.

### 5.4. High-Performance Liquid Chromatography (HPLC)

HPLC can be used to separate and analyze glycan structures following their release from glycoproteins. This method is particularly effective for characterizing complex glycan mixtures and can be coupled with mass spectrometry for detailed analysis [98]. HPLC provides insights into the diversity of glycan structures present on EVs and can help identify specific glycan profiles associated with different physiological or pathological states.

### 5.5. Fluorescent Labeling

Fluorescent labeling techniques can also be employed to study EV glycosylation. By using fluorescently labeled lectins or glycan-binding proteins, it is possible to visualize and quantify glycan expression on the surface of EVs using flow cytometry or fluorescence microscopy [99]. This method allows for assessing glycan distribution and density on EVs, providing insights into their functional roles in cell signaling and intercellular communication.

Despite advancements in EV glycomics, various analytical techniques present specific limitations. Lectin microarrays, though high-throughput and cost-effective, provide semi-quantitative data and are limited by lectin specificity and cross-reactivity [100]. This limitation impacts the accuracy of glycomic profiling, especially when analyzing complex biological samples. Mass spectrometry offers unparalleled structural resolution and specificity but requires complex sample preparation and low throughput and can be cost-prohibitive [101]. Moreover, while enzymatic digestion aids in the validation of glycosylation sites, it may not fully depict complete glycan architecture, thus highlighting a gap in the analytical power of methods used in glycomics [102]. Additionally, HPLC demonstrates superior separation of glycans; however, this method necessitates derivatization steps, which can add complexity and decrease throughput [103]. Fluorescent labeling techniques, which are often utilized for visualizing glycan distribution, also face challenges in quantification due to inconsistencies and a lack of structural detail, further complicating the interpretation of glycomic data in a biological context [104]. The comparative analysis of EV glycosylation techniques is presented in Table 3.

Moreover, the isolation of brain-derived EVs presents considerable technical challenges, particularly due to the low yield stemming from the limited quantity of EVs in cerebrospinal fluid (CSF) and plasma, coupled with the risk of contamination from non-neuronal EVs [105]. The difficulty in differentiating vesicles derived from various CNS cell types intensifies the challenges faced during downstream glycomics analysis. Techniques such as immunoprecipitation utilizing neural-specific markers, like L1CAM and NCAM, have shown promise in enhancing EV enrichment. However, these methods are not entirely specific and often inadvertently co-isolate peripheral vesicles, which can complicate the glycomics analysis process [106].

## 6. NEU1 and EV Glycosylation in Alzheimer’s Disease

Neuraminidase 1 (NEU1) plays a crucial role in modifying the glycosylation patterns of extracellular vesicles (EVs) through its sialidase activity, which involves the enzymatic removal of sialic acid residues from glycoproteins and glycolipids. This modification has significant implications for the properties and functions of EVs in AD. NEU1 is primarily localized in lysosomes but can translocate to the cell surface, where it exerts its sialidase activity [107]. By cleaving terminal sialic acids from glycan structures on the surface of EVs, NEU1 alters the overall glycosylation profile of these vesicles. This desialylation process can influence various aspects of EV biology, including their stability, recognition by recipient cells and subsequent uptake [56,108].

**Figure 4:**
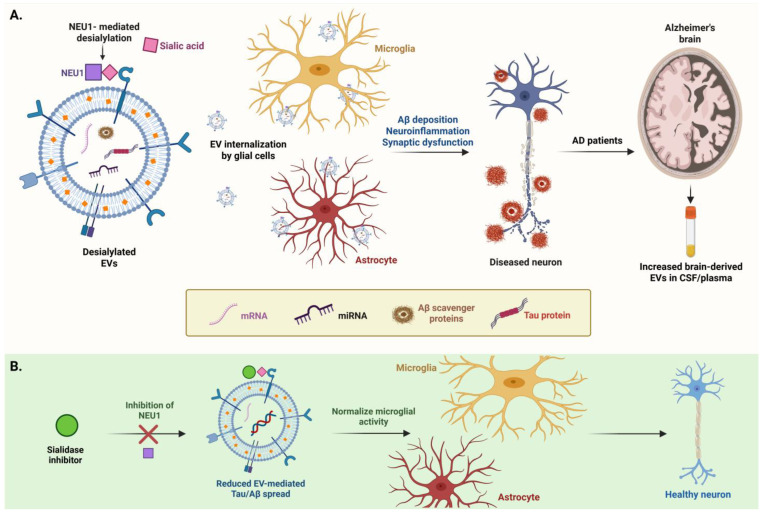
(**A**) In Alzheimer’s disease (AD), NEU1-mediated desialylation decreases the sialic acid content on extracellular vesicles (EVs), altering their surface composition. Microglia and astrocytes take up these desialylated EVs, promoting Aβ deposition, neuroinflammation and synaptic dysfunction, which lead to neurodegeneration. EVs transport mRNA, miRNA, tau and Aβ-related proteins that are found at elevated levels in the cerebrospinal fluid (CSF) and plasma of AD patients. (**B**) Based on current findings, the hypothesis posits that pharmacological inhibition of NEU1 using sialidase inhibitors could preserve EV sialylation, reduce the spread of tau and Aβ and normalize glial cell responses. This intervention may support neuronal survival and maintain brain function in AD patients.

The removal of sialic acids by NEU1 can lead to several changes in EV properties. Sialic acids contribute to the stability of glycoproteins and glycolipids on the EV surface. Their removal can make EVs more susceptible to degradation, potentially affecting their lifespan in circulation [109]. The presence of sialic acids on EVs often serves as a protective mechanism against nonspecific interactions with other cells. By removing these residues, NEU1 can enhance the binding affinity of EVs to specific receptors on target cells, facilitating their uptake [56]. For example, the desialylation of Toll-like receptor 4 (TLR4) by NEU1 has been shown to enhance its signaling capabilities, which may also apply to other receptors involved in EV uptake [108]. The glycan composition of EVs can dictate their interactions with immune cells. For example, the removal of sialic acids can expose underlying glycan structures that may act as ligands for lectins or other glycan-binding proteins, thereby influencing immune responses [110]. This is particularly relevant in the context of neuroinflammation, where EVs can carry pro-inflammatory signals that modulate the activity of microglia and astrocytes (Figure 4) [111].

### 6.1. Implications for Disease Propagation

In the context of AD, NEU1-mediated desialylation of EVs may facilitate the spread of pathological proteins such as Aβ and tau. By altering the glycosylation patterns of EVs, NEU1 can enhance the transfer of these toxic proteins between neurons, contributing to synaptic dysfunction and neurodegeneration [112]. Furthermore, the inflammatory environment created by activated microglia, which can be influenced by NEU1 activity, may further exacerbate the pathological effects of EV-associated proteins [113].

#### 6.1.1. Changes in EV Glycosylation

NEU1 removes sialic acids from EVs, increasing their uptake by neurons. Sialic acids are known to play a protective role on the surface of glycoproteins, preventing non-specific interactions and modulating receptor recognition [114]. The removal of these residues can expose underlying glycan structures, enhancing the binding affinity of EVs to specific receptors on target cells, which facilitates their uptake [115]. For example, integrins, which are often upregulated in inflammatory conditions, can interact more effectively with desialylated EVs, promoting their internalization [89].

#### 6.1.2. Impact on EV Uptake by Neurons

In the context of neurons, the desialylation of EVs can enhance their uptake, potentially leading to increased delivery of pathological proteins such as Aβ and tau [116]. Studies have shown that neurons can internalize EVs through specific receptors that recognize glycan structures, and the presence of sialic acids can inhibit this process [117]. By removing sialic acids, NEU1 may facilitate the transfer of toxic oligomers from EVs to neurons, contributing to synaptic dysfunction and neurodegeneration associated with AD [118].

#### 6.1.3. Influence on Microglial and Astrocytic Uptake

Microglia and astrocytes also play critical roles in the uptake of EVs. NEU1-mediated changes in EV glycosylation can enhance the uptake of these vesicles by activating microglia, which are known to respond to inflammatory signals [115]. The increased presence of integrins and other receptors on the surface of activated microglia can facilitate the internalization of desialylated EVs, leading to the release of pro-inflammatory cytokines and further intensifying neuroinflammation [119]. This inflammatory response can create a feedback loop, where activated microglia release more EVs containing inflammatory mediators, perpetuating the cycle of neuroinflammation and neuronal damage [84]. Astrocytes can also internalize EVs, and NEU1’s activity may influence their functional state. The uptake of EVs by astrocytes can modulate their neuroprotective or neurotoxic effects, depending on the context [120]. For example, astrocytic uptake of EVs containing inflammatory signals can lead to a reactive state that may harm surrounding neurons, while the uptake of EVs with neuroprotective factors can promote neuronal survival [121].

#### 6.1.4. Consequences for Disease Propagation

The changes in EV glycosylation mediated by NEU1 have significant implications for the propagation of AD. By facilitating the transfer of toxic proteins and inflammatory signals through altered EV uptake, NEU1 may contribute to the spread of AD pathology within the brain. This interplay between EVs, NEU1 activity and cellular responses highlights the importance of glycosylation in the pathophysiology of neurodegenerative diseases [122].

## 7. Specific Examples of Glycans Affected by NEU1 and Their Relevance to AD

Neuraminidase 1 (NEU1) significantly influences the glycosylation patterns of EVs, which can alter the sorting and packaging of cargo within these vesicles. This modulation has important implications for the spread of pathological proteins such as Aβ, tau and inflammatory mediators, particularly in AD.

### 7.1. NEU1-Mediated Changes in EV Glycosylation

The sialidase activity of NEU1 removes sialic acid residues from glycoproteins and glycolipids on the surface of EVs. This desialylation alters the overall glycan composition, which can impact the sorting mechanisms that determine what cargo is packaged into EVs. The presence of sialic acids is known to influence the interactions between EVs and their cellular environment, including the binding to specific receptors on target cells [123]. By removing these residues, NEU1 may facilitate the exposure of underlying glycan structures that can enhance the binding affinity of EVs to receptors on recipient cells, thereby influencing the selection of cargo that is incorporated into EVs [124].

### 7.2. Impact on Cargo Sorting and Packaging

The glycosylation status of EVs can directly affect the sorting of proteins and other molecules into these vesicles. Changes in glycosylation patterns mediated by NEU1 may alter the interactions of cargo proteins with sorting machinery in the endosomal system, leading to differential packaging of proteins such as Aβ and tau [125]. For example, desialylated EVs may preferentially incorporate certain proteins that are involved in inflammatory responses or neurodegeneration, thereby enhancing the pathological effects of these cargoes [126].

### 7.3. Influence on the Spread of Aβ and Tau

The altered glycosylation of EVs due to NEU1 activity can facilitate the spread of toxic proteins like Aβ and tau. Studies have shown that EVs containing Aβ oligomers can induce neurotoxicity in recipient neurons, and the efficiency of this process may be influenced by the glycosylation state of the EVs [127]. By promoting the uptake of EVs with altered glycosylation, NEU1 may enhance the transfer of these pathological proteins between cells, contributing to the prion-like propagation of AD pathology [112]. Similarly, tau protein released in EVs can seed aggregation in neighboring neurons, intensifying tau pathology and neurodegeneration [128].

### 7.4. Role of Inflammatory Mediators

NEU1-mediated changes in EV glycosylation can also impact the packaging and release of inflammatory mediators. EVs derived from activated microglia or astrocytes can carry pro-inflammatory cytokines and other signaling molecules that influence the neuroinflammatory environment in AD [129]. By altering the glycosylation patterns of these EVs, NEU1 may enhance their ability to activate neighboring glial cells or neurons, perpetuating the inflammatory response and contributing to neuronal damage [130].

### 7.5. Implications for Therapeutic Strategies

Understanding the role of NEU1 in modulating EV glycosylation and its subsequent effects on cargo sorting and pathology may provide insights into potential therapeutic strategies for AD. Targeting NEU1 activity could help regulate the glycosylation patterns of EVs, potentially mitigating the spread of toxic proteins and the associated inflammatory responses [131,132].

## 8. NEU1, EV Glycosylation and Neuroinflammation in AD

### 8.1. NEU1’s Role in Microglial Activation and Cytokine Release via EV Glycosylation

NEU1 plays a significant role in modulating the glycosylation of EVs released by microglia, which in turn influences microglial activation states and the release of pro- and anti-inflammatory cytokines. The enzymatic activity of NEU1, which involves the removal of sialic acid residues from glycoproteins and glycolipids, has profound implications for the functional properties of EVs and their interactions with recipient cells.

### 8.2. NEU1 and Glycosylation of EVs

The sialidase activity of NEU1 leads to the desialylation of glycoproteins on the surface of EVs released by activated microglia. This process alters the glycan composition of EVs, which can affect their stability, targeting and uptake by recipient cells, including other microglia, neurons and astrocytes [133,134]. The removal of sialic acids exposes underlying glycan structures that can enhance binding to specific receptors on target cells, facilitating the uptake of EVs and their cargo.

### 8.3. Influence on Microglial Activation States

The changes in glycosylation patterns mediated by NEU1 can significantly influence microglial activation states, particularly the polarization between M1 (pro-inflammatory) and M2 (anti-inflammatory) phenotypes. Desialylated EVs may promote M1 polarization by enhancing the activation of Toll-like receptor 4 (TLR4) signaling pathways, which are crucial for initiating inflammatory responses [56]. For instance, NEU1 activity has been shown to desialylate TLR4, leading to enhanced signaling and the subsequent release of pro-inflammatory cytokines such as IL-6 and TNF-α [135]. This M1 polarization is associated with increased neuroinflammation and neurotoxicity, contributing to the progression of AD [136]. Conversely, the glycosylation changes induced by NEU1 may also influence the transition to an M2 phenotype under certain conditions. M2 macrophages are known for their anti-inflammatory properties and ability to promote tissue repair. If NEU1 activity is modulated, it could potentially shift the balance towards M2 polarization by altering the glycan profiles of EVs that interact with microglia, thereby promoting a more neuroprotective environment [137,138].

### 8.4. Cytokine Release and Inflammatory Response

The glycosylation status of EVs has direct implications for the release of cytokines from microglia. Desialylated EVs can enhance the secretion of pro-inflammatory cytokines, thereby exacerbating neuroinflammation in AD [3]. For example, the removal of sialic acids from EVs can lead to increased levels of IL-1β and IL-6, which are critical mediators of the inflammatory response [139]. This inflammatory milieu can further activate neighboring microglia and astrocytes, creating a feedback loop that perpetuates neuroinflammation and neuronal damage [3]. On the other hand, if NEU1 activity is regulated to favor sialylation, it may help maintain a more balanced inflammatory response, reducing the release of pro-inflammatory cytokines and promoting the secretion of anti-inflammatory mediators [77]. This balance is crucial for preventing excessive neuroinflammation, which is a hallmark of AD pathology.

### 8.5. Implications for Alzheimer’s Disease

The influence of NEU1 on EV glycosylation and microglial activation has significant implications for the pathogenesis of AD. By modulating the glycosylation patterns of EVs, NEU1 can affect the spread of pathological proteins such as Aβ and tau, as well as the inflammatory responses that contribute to neurodegeneration [140]. Understanding these mechanisms may provide insights into potential therapeutic strategies aimed at targeting NEU1 activity to regulate microglial function and mitigate the progression of AD. Therefore, NEU1’s role in modifying the glycosylation of EVs released by microglia has profound effects on microglial activation states and the release of cytokines. By influencing the balance between M1 and M2 polarization, NEU1-mediated changes in EV glycosylation can contribute to the inflammatory environment characteristic of AD, highlighting the potential for NEU1 as a therapeutic target.

### 8.6. NEU1 and Astrocyte-Derived EV Glycosylation

The sialidase activity of NEU1 leads to the removal of sialic acid residues from glycoproteins and glycolipids on the surface of astrocyte-derived EVs. This desialylation alters the glycosylation patterns of these vesicles, which can influence their stability, targeting and uptake by recipient cells, including microglia and neurons [141,142]. The loss of sialic acids can enhance the binding of EVs to specific receptors on target cells, facilitating their internalization and subsequent effects on cellular signaling pathways [141].

### 8.7. Influence on Microglial Activation

The changes in glycosylation mediated by NEU1 can significantly impact the activation states of microglia. Desialylated EVs may promote the M1 (pro-inflammatory) polarization of microglia, leading to increased production of pro-inflammatory cytokines such as IL-1β and TNF-α [87,143]. This M1 polarization is associated with a neurotoxic environment that exacerbates neuroinflammation and contributes to neuronal damage in AD [143]. Conversely, if NEU1 activity is regulated to favor sialylation, it may help maintain a more balanced inflammatory response, potentially promoting M2 (anti-inflammatory) polarization and neuroprotection [87].

### 8.8. Cytokine Release and Neuroinflammation

Astrocyte-derived EVs can carry various cytokines and signaling molecules that influence the inflammatory response in the brain. NEU1-mediated changes in glycosylation can affect the cargo composition of these EVs, leading to the differential release of pro-inflammatory or anti-inflammatory factors [132]. For instance, EVs containing IL-1β can enhance the activation of microglia, promoting a cascade of inflammatory responses that contribute to the pathology of AD [141]. The interaction between NEU1 activity, EV glycosylation and cytokine release underscores the importance of astrocytic EVs in modulating neuroinflammation.

### 8.9. Implications for Aβ and Tau Pathology

The glycosylation changes in astrocyte-derived EVs influenced by NEU1 may also impact the spread of pathological proteins such as Aβ and tau. Studies have shown that EVs can carry Aβ oligomers and tau, facilitating their transfer between cells and contributing to the propagation of neurodegenerative processes [144,145]. By altering the glycan composition of these EVs, NEU1 may enhance the transfer of toxic proteins, exacerbating synaptic dysfunction and neurodegeneration associated with AD [146].

## 9. Therapeutic Implications of Targeting NEU1 in AD

The modulation of neuraminidase 1 (NEU1) activity presents a promising therapeutic strategy for altering extracellular vesicle (EV) glycosylation and potentially mitigating the pathology of AD. NEU1 is known to play a crucial role in the desialylation of glycoproteins, influencing various cellular processes, including intercellular communication and disease propagation. Given its involvement in the regulation of glycosylation patterns on EVs, targeting NEU1 could provide a novel approach to address the dysregulated sialylation observed in AD.

One potential therapeutic strategy involves the use of transmembrane peptides designed to inhibit NEU1 activity. Such peptides have been shown to effectively block NEU1 activation, which could lead to an increase in sialylation levels on EVs, thereby enhancing their protective roles in neuronal communication and possibly reducing amyloid-β accumulation [147]. This approach aligns with findings that NEU1 deficiency results in an oversialylated state for the amyloid precursor protein (APP), leading to increased amyloid-β secretion [55]. By restoring appropriate sialylation levels through NEU1 inhibition, it may be possible to modulate the release and composition of EVs, thereby influencing AD pathology.

**Figure 5:**
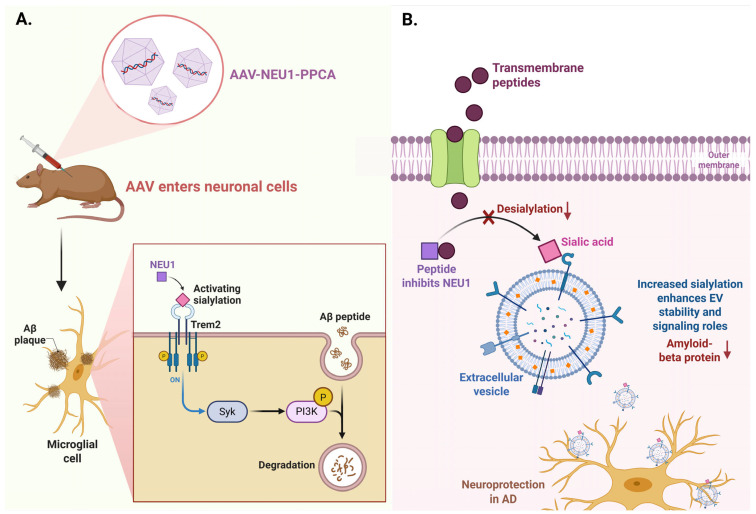
(**A**) Gene therapy using AAV-NEU1-PPCA delivers NEU1 and its chaperone PPCA into neuronal cells, enhancing NEU1 activity in microglia. This regulates the sialylation of the TREM2 receptor, activates Syk-PI3K signaling and promotes amyloid-β degradation, thereby supporting microglial function. (**B**) Transmembrane peptides inhibit NEU1-mediated desialylation at the membrane, increasing the sialylation of extracellular vesicles (EVs). Enhanced sialylation stabilizes EVs, improves their signaling roles and reduces amyloid-β levels, contributing to neuroprotection in AD.

Another promising avenue is the application of gene therapy techniques targeting NEU1. For instance, chaperone-mediated gene therapy using recombinant adeno-associated virus (AAV) vectors to deliver NEU1 or its chaperone, PPCA, has shown potential in animal models of lysosomal storage diseases [148]. This strategy could be adapted to enhance NEU1 expression in neuronal cells, potentially correcting the dysregulated glycosylation patterns that contribute to AD. Furthermore, enhancing NEU1 activity in specific contexts, such as in microglia, may help regulate the sialylation of receptors like Trem2, which is critical for microglial function and neuroinflammation (Figure 5) [149].

Additionally, pharmacological agents that modulate NEU1 activity could be explored. For instance, compounds that enhance NEU1 function have been linked to improved insulin signaling and metabolic outcomes, suggesting that similar strategies could be applied to neuronal cells to restore normal EV glycosylation and function [150]. The interplay between NEU1 and various signaling pathways, including those involved in inflammation and cellular stress responses, highlights the potential for targeted pharmacological interventions to recalibrate NEU1 activity in the context of neurodegenerative diseases [57]. In summary, therapeutic strategies targeting NEU1 to modulate EV glycosylation hold significant promise for addressing the underlying mechanisms of AD. By employing peptide inhibitors, gene therapy and pharmacological agents, it may be possible to restore normal glycosylation patterns, enhance intercellular communication and mitigate the progression of AD pathology (Table 4).

However, recent therapeutic advancements targeting NEU1 face substantial translational challenges, one of which is the hindrance posed by the blood–brain barrier (BBB). The restrictive nature of the BBB limits the efficacy of systemically administered NEU1 inhibitors in accessing the CNS [151,152]. To address this challenge, advanced nanocarrier platforms such as liposomes, polymeric nanoparticles and engineered extracellular vesicles (EVs) have been proposed as efficient delivery systems. These nanocarriers can be designed with targeting ligands (transferrin, RVG peptides) to promote receptor-mediated transport across the BBB, optimizing the delivery of NEU1 inhibitors or gene-editing agents specifically to neuronal and glial cells [153,154,155]. EVs, in particular, exhibit beneficial characteristics, including biocompatibility, low immunogenicity and natural affinity for brain tissue, making them compelling candidates for CNS-targeted therapies [155].

Furthermore, isoform specificity of NEU1 inhibitors poses another crucial hurdle in therapeutic development. Existing inhibitors often lack the ability to selectively target NEU1 as opposed to other sialidase isoforms such as NEU2, NEU3 and NEU4, which increases the risk for undesirable systemic effects [110,156]. Given that NEU1 contributes to essential physiological functions, such as lysosomal activity, insulin signaling and immune response regulation, systemic inhibition may inadvertently disrupt these pathways, leading to off-target effects [112,157,158]. Reported studies have illustrated that the modulation of NEU1 can have profound implications for insulin receptor activation, thereby influencing metabolic pathways critical in conditions such as obesity and diabetes [150,157]. Therefore, achieving precise control over NEU1 activity through methodologies that focus on cell type-specific delivery, temporal regulation and comprehensive safety profiling is important for the successful clinical translation of NEU1-targeted interventions in diseases like AD [58].

**Table 4:** Therapeutic approaches targeting NEU1 in AD pathogenesis.

Therapeutic Strategy	Description	Mechanism of Action	References
Chemical Inhibitors	DANA (2,3-dehydro-2-deoxy-N-acetylneuraminic acid)	Broad-spectrum inhibition of NEU1 and related isoenzymes. Enhances LFA-1 adhesion, potentially improving synaptic plasticity issues in AD models	[141,159,160]
Transmembrane Peptides	Interfering peptides designed to block NEU1 dimerization	Disruption of NEU1 activation, potentially reducing sialic acid-mediated signaling that contributes to neural impairment	[147]
Elastin-Derived Peptides	Peptides activating NEU1	Induce NEU1 activity leading to alterations in ganglioside profiles, which can influence inflammation and neuronal communication	[161,162]
Combination Therapies	Multi-target approaches combining NEU1 inhibitors with other pathways	Synchronously targeting NEU1 alongside BACE-1 or inflammatory pathways to provide a synergistic effect in mitigating AD pathology	[163,164]
Biomarkers and Diagnostics	Monitoring of sialylation changes as potential early indicators	Tracking NEU1 activity to assess the progression of neurodegeneration, aiding in early intervention strategies	[109,134]

(DANA—2,3-dehydro-2-deoxy-N-acetylneuraminic acid, LFA-1—Lymphocyte function-associated antigen 1, BACE-1—Beta-site APP cleaving enzyme 1.)

## 10. NEU1 Inhibitors

The exploration of neuraminidase 1 (NEU1) inhibitors has gained significant advancements, particularly regarding a therapeutic strategy for various diseases, including AD and other degenerative conditions. NEU1 plays a crucial role in modulating sialic acid residues on glycoproteins, which influences critical biological processes such as cellular communication and immune responses [56,117]. The development of selective NEU1 inhibitors presents a promising approach that could afford valuable therapeutic benefits by modulating these processes while avoiding the off-target effects associated with broader-spectrum agents. Existing NEU1 inhibitors, such as DANA (N-Acetyl-2,3-dehydro-2-deoxyneuraminic acid) and zanamivir, are effective. However, they are not selective and can inhibit other neuraminidases, including NEU3 [165,166]. This lack of specificity has significant implications, as studies have indicated that nonspecific inhibition can lead to unwanted side effects, which compromise therapeutic efficacy [59,167,168]. Consequently, there is a pressing need for selective NEU1 inhibitors that could mitigate these adverse effects while retaining therapeutic potential.

One noteworthy example of a selective NEU1 inhibitor is CG33300, which has demonstrated significant efficacy in preclinical models of pulmonary inflammation and fibrosis. This compound selectively inhibits NEU1 activity without significantly affecting other neuraminidases, thus minimizing off-target effects and enhancing therapeutic specificity [165,169]. Additionally, C9-butyl-amide-DANA has emerged as another selective inhibitor that effectively blocks NEU1 activity in human lung endothelial cells and fibroblasts, showcasing the potential of these molecules to modulate inflammatory responses effectively [127,170]. The therapeutic utility of these selective inhibitors highlights their potential in various disease contexts linked to NEU1 dysregulation, particularly in modulating inflammatory processes.

Furthermore, recent advancements have led to the development of novel inhibitors based on the DANA scaffold, with enhancements at positions C5 and C9 to improve selectivity and potency specifically against NEU1 [57,165,171]. These innovations have been validated through both in vitro and in vivo studies that confirm their ability to reduce NEU1 activity effectively. The specificity of these inhibitors is vital, as it allows for targeted modulation of the effects of NEU1 on glycosylation and cellular signaling pathways, avoiding disruption of the functions of other neuraminidases and potentially leading to more favorable clinical outcomes [107,172]. The therapeutic implications of NEU1 inhibition extend beyond inflammation and fibrosis. Recent investigations show the role of NEU1 in regulating insulin receptor activity, where its inhibition can enhance insulin signaling, providing a promising intervention for metabolic disorders such as diabetes [58]. Additionally, NEU1 is involved in critical immune regulatory functions. Thus, its selective inhibition may enhance phagocytic activity in macrophages and microglia, which is particularly noteworthy in the context of neurodegenerative diseases like AD [107,168,173]. Given the multifaceted roles of NEU1 in various biological processes, the strategic targeting of this enzyme through selective inhibitors represents an innovative and promising therapeutic strategy for managing pathological conditions associated with dysregulated sialylation.

The interplay between NEU1 activity, sialylation and amyloid precursor protein (APP) metabolism further highlights its therapeutic potential in neurodegenerative disorders. Studies indicate that a loss of NEU1 function results in the accumulation of hypersialylated APP, leading to increased amyloidogenic processing and the release of amyloid-beta peptides pivotal in the pathogenesis of AD [107,165,167,168,171]. In this regard, targeted NEU1 inhibition could address multiple aspects of AD pathology, combining anti-inflammatory effects with a potential reduction in amyloid-beta burden through modulation of APP metabolism and sialylation status [57,107,168].

Moreover, the various NEU1 inhibitors in development, including those based on DANA scaffolding, highlight the imperative for ongoing therapeutic exploration within this domain. Compounds like CG33300 and C9-butyl-amide-DANA emphasize the necessity for specificity and selectivity in NEU1 modulating agents that may alleviate or prevent the pathological processes linked with a multitude of diseases, notably those associated with neurodegeneration and chronic inflammatory states [57,127,169]. The versatility of NEU1-targeted therapies also suggests avenues for future research, potentially expanding their application to a broader array of conditions characterized by aberrant glycosylation processes or cellular signaling dysfunction [56,57,58,117,127,169,174].

The increasing evidence supporting the therapeutic potential of NEU1 inhibitors positions them as an important agent in advancing treatment paradigms for complex diseases such as Alzheimer’s and metabolic disorders. The development of selective NEU1 inhibitors that maintain efficacy while minimizing off-target effects reflects an essential stride in achieving targeted therapeutic outcomes. Using the unique properties of NEU1 and its inhibitors holds significant promise for enhancing treatment landscapes across diverse pathological conditions, which could significantly improve patient outcomes and quality of life for those affected by such debilitating diseases.

## 11. Glycan Engineering Approaches

Glycan engineering approaches for modifying extracellular vesicle (EV) glycosylation are gaining attention due to their potential to enhance the functionality and therapeutic applications of EVs. These strategies involve various techniques aimed at altering the glycan composition on the surface of EVs, which can significantly influence their biological properties, including cellular uptake, immune modulation and intercellular communication.

One prominent approach involves the genetic engineering of EV-associated proteins to display specific glycan structures. For instance, Zheng et al. (2022) demonstrated the conversion of CD63, a well-characterized tetraspanin protein, into a carrier for the sialyl Lewis X (sLeX) glycan, which is known to enhance cell adhesion and targeting [175]. By utilizing the large extracellular loop of CD63, researchers can effectively display functional moieties that can be recognized by specific receptors on target cells, thereby improving the targeting efficiency of EVs. This method highlights the potential of using glycan engineering to create EVs with tailored surface properties for specific therapeutic applications.

Another innovative strategy is the use of lipidic tags and viral scaffolds to facilitate the incorporation of glycosylated proteins into the lumen of EVs. This approach allows for the maintenance of the EV’s sorting capacity while enabling the display of peptides or proteins on its extracellular loops [176]. Such modifications can enhance the therapeutic efficacy of EVs by allowing them to deliver specific cargoes more effectively to target cells.

Additionally, the manipulation of glycosyltransferases has emerged as a viable strategy to directly modify the glycan profiles of EVs. For example, Clark et al. investigated the impact of increased fucosyltransferase 8 (FUT8) expression on the EV proteome in prostate cancer cells, revealing that altered glycosylation patterns, which affect protein stability, may influence EV functionality [90]. By upregulating specific glycosyltransferases, researchers can create EVs with distinct glycan signatures that may enhance their interaction with target cells and modulate immune responses.

Moreover, the isolation techniques employed can influence the glycosylation profiles of EVs. Freitas et al. highlighted that different isolation methods lead to diverse glycosylated EV populations, emphasizing the importance of selecting appropriate techniques to achieve the desired glycan modifications [177]. This aspect is critical for ensuring that the engineered EVs possess the intended biological characteristics for therapeutic applications.

The incorporation of glycan engineering into EV research also extends to understanding the role of glycans in cellular uptake. Williams et al. discussed how modifications to glycan structures can reduce steric hindrance, thereby facilitating the interaction of EV surface ligands with cell surface receptors [178]. This insight underscores the potential for glycan engineering to enhance the biodistribution and efficacy of EV-based therapies. Hence, glycan engineering approaches for modifying EV glycosylation encompass a range of strategies, including genetic modifications, glycosyltransferase manipulation and optimized isolation techniques. These methods hold significant promise for enhancing the therapeutic potential of EVs by improving their targeting capabilities, modulating immune responses and facilitating cellular uptake. As research in this area continues to evolve, the development of tailored EVs with specific glycan profiles may lead to novel therapeutic interventions for various diseases, including cancer and neurodegenerative disorders.

## 12. Combination Therapies Targeting NEU1 and Other AD Pathways

The potential for combination therapies targeting neuraminidase 1 (NEU1) alongside other AD pathways presents an innovative approach to managing this complex neurodegenerative disorder. Given the multifactorial nature of AD, integrating NEU1-targeting strategies with existing treatments could enhance therapeutic efficacy and address various pathological mechanisms simultaneously.

**Figure 6:**
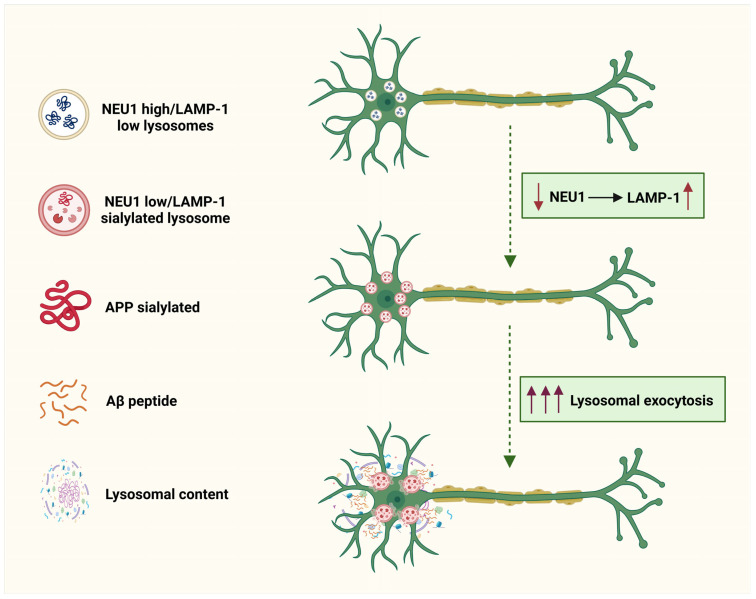
Lysosomal NEU1 deficiency drives lysosomal exocytosis and enhances the sialylation of amyloid precursor protein (APP) in neurons. Under physiological conditions, elevated NEU1 expression and low LAMP-1 levels regulate lysosomal homeostasis, preventing excessive accumulation. However, NEU1 downregulation leads to upregulation of LAMP-1, increased lysosomal sialylation and aberrant APP sialylation. This disruption promotes lysosomal exocytosis, facilitating the extracellular release of lysosomal content and Aβ peptides implicated in amyloid pathology.

One promising avenue for combination therapy involves the modulation of NEU1 activity to influence amyloid precursor protein (APP) processing and amyloid-β (Aβ) secretion. Research has shown that NEU1 deficiency leads to increased lysosomal exocytosis, which may facilitate the release of Aβ from lysosomes [55] (Figure 6). By inhibiting NEU1, it may be possible to reduce the extracellular release of Aβ, thereby complementing existing anti-amyloid therapies. This strategy could be particularly effective when combined with agents that target amyloid aggregation or promote clearance, such as monoclonal antibodies against Aβ [148]. The dual approach of inhibiting NEU1 while enhancing Aβ clearance could synergistically reduce amyloid burden and improve cognitive outcomes.

Another potential combination strategy involves the use of gene therapy to restore NEU1 function alongside treatments that target tau pathology. For instance, the use of adeno-associated virus (AAV) vectors to deliver NEU1 or its chaperone, PPCA, could be combined with tau-targeting therapies, such as those that inhibit tau aggregation or promote tau clearance through microglial activation [65]. This dual targeting could address both amyloid and tau pathologies, which are critical components of AD progression.

Additionally, the role of extracellular vesicles (EVs) in AD pathology opens up further avenues for combination therapies. EVs are known to facilitate the spread of toxic proteins, including tau and Aβ, within the brain [179]. By engineering EVs to carry therapeutic agents that target both NEU1 and tau or Aβ, it may be possible to enhance the delivery of these agents directly to affected neurons. For example, modifying EVs to express specific ligands that bind to neuronal receptors could improve their uptake and therapeutic efficacy [180]. This approach aligns with the concept of using EVs as drug delivery vehicles, which has been explored in various preclinical models [181].

Moreover, the interplay between NEU1 and other metabolic pathways, such as sphingolipid metabolism, suggests that targeting these pathways in conjunction with NEU1 inhibition could yield beneficial effects. Alterations in sphingolipid metabolism have been implicated in AD, and combining NEU1-targeting therapies with agents that modulate sphingolipid levels may enhance neuroprotection and reduce neuroinflammation [182]. Therefore, the integration of NEU1-targeting therapies with other AD treatments holds significant promise for developing more effective therapeutic strategies. By addressing multiple pathways involved in AD pathology, such as amyloid and tau aggregation, lysosomal function and EV-mediated protein spread, combination therapies could provide a comprehensive approach to mitigating disease progression and improving patient outcomes.

The exploration of glycosylation patterns of EVs presents a fascinating area for diagnosing and prognosing AD. Glycans, due to their sensitivity to cellular metabolism and disease states, emerge as significant biomarkers reflecting alterations in disease processes [183]. In AD, the enzyme NEU1 is linked to changes in glyco-signatures of EVs. Specifically, aberrant activity of NEU1 can lead to variations in terminal sialylation levels, which may serve as indicators of disease activity in AD [183]. Recent studies indicate that EVs derived from the cerebral site, particularly those isolated from cerebrospinal fluid (CSF) and plasma, exhibit distinctive alterations in glycan composition among AD patients. This includes an increase in specific glycan residues potentially attributed to NEU1-mediated desialylation [184]. Utilization of advanced methodologies such as lectin microarrays, mass spectrometry and glycan-specific probes facilitates the detection of these surface glycan changes, enabling the establishment of EV profiles closely associated with the pathology of AD [185]. This data aligns with the literature, suggesting that the signature glycosylation patterns of EVs can act as disease biomarkers [183].

Despite the promising role that EV glycosylation may play as a biomarker for AD, several challenges hinder its clinical implementation. One primary concern is specificity; glycan changes occurring in AD may also overlap with those seen in other neurodegenerative diseases, complicating differential diagnosis [185]. Furthermore, the heterogeneous nature of EV populations complicates the standardization of isolation techniques necessary for obtaining CNS-specific vesicles, which is critical for accurate biomarker analysis [177]. The variations in EV isolation protocols and detection methods can impact the reproducibility of findings across various studies, highlighting the need for standardization in EV research [185].

Furthermore, validation remains pivotal, as many studies to date have operated on limited sample sizes, indicating a critical need for multicenter trials and longitudinal studies to enhance clinical validation efforts [186]. Nonetheless, the potential to utilize EV glycosylation as a minimally invasive biomarker for early diagnosis, progression monitoring and patient stratification in AD is significant. Future actions should hold rigorous standardization practices, enhance CNS-EV enrichment techniques and establish glyco-signature panels that are validated across diverse patient populations to facilitate the clinical translation of these biomarkers [177].

## 13. Future Directions and Challenges

The exploration of NEU1 in the context of AD presents several key research questions and unresolved issues that warrant further investigation. Understanding the precise mechanisms by which NEU1 regulates extracellular vesicle (EV) glycosylation and its downstream effects is critical for elucidating its role in AD pathology. Research indicates that NEU1 influences the sialylation of glycoproteins, which can affect cellular communication and the propagation of neurodegenerative processes [58]. However, the specific pathways and molecular interactions involved in NEU1-mediated glycosylation changes remain poorly understood. Future studies should aim to delineate these mechanisms to better comprehend how NEU1 contributes to the pathophysiology of AD. To strengthen the mechanistic understanding, future research should prioritize key questions such as

- Does NEU1 knockdown in vivo reduce EV-mediated Aβ or tau propagation in AD models?
- What specific glycoproteins on EVs are regulated by NEU1, and how do their desialylated forms affect cellular uptake by neurons, astrocytes or microglia?
- Can EV-associated glycosylation profiles serve as early biomarkers to predict AD onset or therapeutic response?
- How does NEU1 activity vary across brain regions and disease stages, and what are the regional implications for EV function and pathology?

Another area of focus is the development of more specific NEU1 inhibitors. Current inhibitors, such as DANA, lack selectivity and can inhibit multiple neuraminidase isoforms, leading to potential off-target effects [187]. The identification of selective NEU1 inhibitors could enhance therapeutic strategies by minimizing adverse effects associated with broader inhibition. Advances in structural biology, including the recent elucidation of NEU1’s three-dimensional structure, may facilitate the rational design of specific inhibitors [115]. This could lead to the development of novel compounds that selectively modulate NEU1 activity without affecting other neuraminidases, thereby improving the safety and efficacy of NEU1-targeting therapies.

Beyond therapeutic development, EV glycosylation profiling represents a promising tool for AD diagnosis. Altered glycosylation patterns in brain-derived EVs may reflect NEU1 activity and disease severity, offering a non-invasive biomarker for clinical use. Integration of EV glycomics with other omics approaches (proteomics and transcriptomics) could uncover new pathways involved in early AD pathogenesis and progression. The potential of using EV glycosylation as a biomarker for AD also represents a promising research direction. Alterations in the glycosylation patterns of EVs have been implicated in various diseases, including neurodegenerative disorders [110,188]. Investigating the relationship between NEU1 activity, EV glycosylation and AD progression could yield valuable insights into the disease’s pathophysiology and aid in the identification of novel biomarkers for early diagnosis and monitoring of therapeutic responses.

Despite the promising avenues for NEU1-targeting therapies, several challenges remain. One significant challenge is ensuring specificity in targeting NEU1 to avoid off-target effects that could arise from inhibiting other neuraminidases. The overlapping functions of different neuraminidase isoforms complicate the development of selective inhibitors, necessitating a thorough understanding of the unique roles of NEU1 in various cellular contexts [57].The delivery of NEU1-targeting therapies to the brain poses another challenge. The blood–brain barrier (BBB) presents a significant obstacle for the effective delivery of therapeutic agents, including small molecules and biologics. Innovative delivery systems, such as nanoparticles or EVs engineered to cross the BBB, may be required to enhance the bioavailability of NEU1 inhibitors in the central nervous system [189]. Innovative strategies such as nanoparticle-based delivery, glycan-functionalized ligands or engineered EVs capable of BBB crossing may offer targeted solutions. Endogenous EV pathways could also serve as delivery vehicles for NEU1-specific RNA interference or CRISPR-based modulation.

Overall, future research should focus on elucidating the mechanisms by which NEU1 regulates EV glycosylation, developing specific NEU1 inhibitors and exploring the potential of EV glycosylation as a biomarker for AD. Addressing the challenges of specificity and delivery will be crucial for advancing NEU1-targeting therapies and improving outcomes for individuals affected by AD.

## 14. Conclusions

The present review highlights the critical role of neuraminidase 1 (NEU1) in the regulation of extracellular vesicle (EV) glycosylation and its implications for AD pathology. NEU1’s involvement in the desialylation of glycoproteins significantly influences cellular communication, immune responses and the propagation of neurodegenerative processes. The evidence suggests that targeting NEU1 could provide a novel therapeutic strategy to modulate EV glycosylation, potentially mitigating the accumulation of amyloid-β and tau proteins, which are central to AD pathology. The review underscores the necessity for further research to unravel the complex interplay between NEU1, EV glycosylation and AD pathogenesis. Key areas for future investigation include elucidating the precise mechanisms by which NEU1 regulates glycosylation and the downstream effects on neuronal health and function. Additionally, the development of more specific NEU1 inhibitors is crucial to minimize off-target effects and enhance therapeutic efficacy. The potential of using altered EV glycosylation as a biomarker for AD also warrants exploration, as it could facilitate early diagnosis and monitoring of disease progression. Despite the promising prospects of NEU1-targeting therapies, challenges remain in ensuring specificity and effective delivery to the brain. The overlapping functions of different neuraminidase isoforms complicate the development of selective inhibitors, necessitating a thorough understanding of NEU1’s unique roles in various cellular contexts. Moreover, innovative delivery systems will be essential to overcome the blood–brain barrier and ensure that therapeutic agents reach their intended targets within the central nervous system. In summary, NEU1 emerges as a vital therapeutic target in AD, with the potential to influence disease progression through its regulatory effects on EV glycosylation. Continued research in this area is essential to fully harness the therapeutic potential of NEU1 and to develop effective strategies for combating AD. While current therapies for AD primarily focus on reducing Aβ or tau pathology, such as monoclonal antibodies (aducanumab and lecanemab) and tau aggregation inhibitors, these approaches have shown limited efficacy and are often associated with adverse effects. In contrast, targeting NEU1 offers a novel strategy by modulating EV glycosylation, which may indirectly affect both amyloid and tau dynamics while also influencing broader neuroinflammatory and intercellular communication pathways. Thus, NEU1-targeted therapies may complement or enhance the effectiveness of existing treatments, providing a multifaceted approach to AD management.

## Figures and Tables

**Table 2:** Role of EVs in AD pathogenesis and diagnostic applications.

EV Source	Cargo	Functional Role in AD Progression or Diagnosis	References
Endothelial cells	Amyloid-beta (Aβ)	Facilitate the translocation of neurotoxic Aβ peptides across the blood–brain barrier (BBB), contributing to AD pathology	[71]
Brain-derived EVs	Aβ, tau, glial markers (ANXA5, VGF, GPM6A, ACTZ)	Transport pathological proteins and glial-specific markers; may serve as diagnostic indicators for AD	[72]
Neurons	Aβ	Mediate inter-neuronal propagation of Aβ in a prion-like fashion or from neurons to microglia, exacerbating neurodegeneration	[61,73]
Neurons	Aβ, tau, mRNA, miRNA	EV-associated nucleic acids and proteins can be detected in plasma/serum, useful as potential blood-based biomarkers	[73,74,75,76]
Neurons	Tau	Pathological tau proteins in cerebrospinal fluid (CSF)-derived EVs are candidate biomarkers for early AD diagnosis	[77]
Astrocytes	Aβ	Transfer of Aβ from astrocytes to neurons, promoting intracellular Aβ accumulation	[78]
Astrocytes	Inflammatory mediators	EVs enriched in proinflammatory proteins serve as blood/plasma biomarkers indicative of neuroinflammation in AD	[79]
Astrocytes	Aβ and tau	The presence of circulating EVs suggests diagnostic utility for disease staging	[74,79,80]
Microglia	Aβ	Enable intercellular communication between microglia and neurons, promoting Aβ dissemination	[64,73,81]
Microglia	Tau	Drive the spread of tau pathology to adjacent neural cells, contributing to tauopathy	[70]

(BBB—Blood–brain barrier, CSF—Cerebrospinal fluid, ANXA5—Annexin A5, GPM6A—Glycoprotein M6A, ACTZ—Alpha-centractin, mRNA—Messenger ribonucleic acid, miRNA—MicroRNA.)

**Table 3:** Comparative analysis of EV glycosylation techniques.

Technique	Resolution	Throughput	Cost	Strengths	Limitations	References
Lectin microarrays	Low–moderate	High	Low–moderate	High-throughput; broad glycan	Cross-reactivity; semi-quantitative	[100]
Mass spectrometry	High (structural)	Low–moderate	High	High specificity; structural glycan mapping	Requires expertise; low throughput; expensive instrumentation	[101]
Enzymatic digestion	Site specific	Low	Low	Identifies glycosylation sites	Limited structural information; not comprehensive	[102]
HPLC	High (separation)	Moderate	Moderate–high	Resolves glycan isomers; suitable for complex mixtures	Requires glycan release/derivatization; moderate throughput	[103]
Fluorescent labeling	Low–moderate	Moderate	Low–moderate	Allows visualization and quantification	Lacks structural resolution; variability in quantification	[104]
